# New Therapies and Strategies to Curb HIV Infections with a Focus on Macrophages and Reservoirs

**DOI:** 10.3390/v16091484

**Published:** 2024-09-18

**Authors:** Maria Marra, Alessia Catalano, Maria Stefania Sinicropi, Jessica Ceramella, Domenico Iacopetta, Romina Salpini, Valentina Svicher, Stefania Marsico, Stefano Aquaro, Michele Pellegrino

**Affiliations:** 1 Department of Pharmacy, Health and Nutritional Sciences, University of Calabria, Via Pietro Bucci, 87036 Arcavacata di Rende, Italy; mariamarra1997@gmail.com (M.M.); s.sinicropi@unical.it (M.S.S.); jessica.ceramella@unical.it (J.C.); domenico.iacopetta@unical.it (D.I.); stefania.marsico@unical.it (S.M.); stefano.aquaro@unical.it (S.A.); michele.pellegrino@unical.it (M.P.); 2 Department of Pharmacy—Drug Sciences, University of Bari “Aldo Moro”, 70126 Bari, Italy; 3 Department of Experimental Medicine, University of Tor Vergata, 00133 Rome, Italy; romina.salpini@uniroma2.it (R.S.); valentina.svicher@uniroma2.it (V.S.)

**Keywords:** cART, HAART, antiretroviral therapy, HIV, AIDS, macrophages, HIV reservoirs, reverse transcriptase inhibitors, protease inhibitors, entry inhibitors, integrase inhibitors

## Abstract

More than 80 million people worldwide have been infected with the human immunodeficiency virus (HIV). There are now approximately 39 million individuals living with HIV/acquired immunodeficiency syndrome (AIDS). Although treatments against HIV infection are available, AIDS remains a serious disease. Combination antiretroviral therapy (cART), also known as highly active antiretroviral therapy (HAART), consists of treatment with a combination of several antiretroviral drugs that block multiple stages in the virus replication cycle. However, the increasing usage of cART is inevitably associated with the emergence of HIV drug resistance. In addition, the development of persistent cellular reservoirs of latent HIV is a critical obstacle to viral eradication since viral rebound takes place once anti-retroviral therapy (ART) is interrupted. Thus, several efforts are being applied to new generations of drugs, vaccines and new types of cART. In this review, we summarize the antiviral therapies used for the treatment of HIV/AIDS, both as individual agents and as combination therapies, and highlight the role of both macrophages and HIV cellular reservoirs and the most recent clinical studies related to this disease.

## 1. Introduction

Acquired immunodeficiency syndrome (AIDS) is a worldwide pandemic currently affecting approximately 39 million people globally [1]. It is caused by two viruses belonging to the *Lentivirus* genus (*Retroviridae* family, *Orthoretrovirinae* subfamily), namely Human Immunodeficiency Virus-1 (HIV-1) and Human Immunodeficiency Virus-2 (HIV-2), which were derived from multiple cross-species transmissions of the Simian Immunodeficiency Viruses (SIVs) before they affected humans. HIV-1 was first discovered in the 1980s [2,3]. However, some studies report that the virus was already present in Africa in the 1950s and in America in the 1970s and spread all over the world in the following years [4]. A few years after its discovery, HIV-1 was shown to be the cause of acquired immune deficiency syndrome (AIDS) [5,6,7]. According to the most recent data from the Joint United Nations Programme on HIV/AIDS (UNAIDS), currently there are approximately 39 million people living with HIV/AIDS worldwide, with 1.3 million people becoming newly infected with HIV in the same year [8]. Although the primary target of HIV infection is represented by CD4+ T cells, the virus also has the ability to infect myeloid cells such macrophages and monocytes (albeit with lower efficiency), since both cells express the HIV primary receptor CD4 and the chemokine co-receptor CCR5, mediating HIV entry [9,10]. The replication of HIV in macrophages involves particular features and differs in many aspects from that in CD4 T lymphocytes. Firstly, HIV shows a different tropism for CD4 T cells and macrophages. Moreover, HIV can also enter macrophages via different unconventional routes, such as the phagocytic uptake of HIV-1-infected CD4+ T cells, cell-to-cell fusion between T cells and macrophages or even by viral spread from HIV infected cells to macrophages by hijacking physiological membrane nanotube structures [11,12]. Lastly, the assembly of viral particles in macrophages is peculiar since it occurs in internal virus-containing compartments, distinct from endosomes and multivescicular bodies, connected to the exterior of the cells by a thin channel, and is thus continuous with the plasma membrane [13,14]. Nevertheless, it should be taken into account that macrophages constitute a less favourable environment for HIV replication and release in respect of the highly proliferative activated CD4 T cells, mostly due to their non-dividing and differentiated state and the presence of host cell restriction factors [15,16].

Given their functions in host defence against pathogens and the regulation of the immune response plus their permissivity to HIV-1 infection, monocytes/macrophages or monocyte-derived macrophages (MDM) exert a dual role in HIV infection. On one hand, they promote HIV-1 persistence and pathogenesis by activating surrounding T cells (a process favouring the establishment of productive HIV infection) and mediating HIV dissemination to other body compartments, such as the central nervous system, since the early phases of HIV infection [17,18,19]. On the other hand, MDM exert an antiviral activity by expressing several molecules that inhibit HIV-1 replication. In particular, activated microglia and macrophages may also have a neurotrophic and neuroprotective effect on the infected brain regulating glutamate metabolism or by the secretion of neurotrophins [20].

To date, blood monocytes and tissue macrophages are thus considered important cellular reservoirs for HIV persistence, together with latently infected CD4+ T cells, representing a relevant source of viral rebound following ART cessation. Moreover, their increased resistance to HIV-induced cytopathic effects and reduced susceptibility to some antiretroviral drugs may also be relevant to residual replication under ART [10,21,22].

On these bases, treating HIV/AIDS is a critical but challenging clinical goal. Almost every AIDS patient died before the mid-1990s. AIDS was the primary killer disease in the US in 1993. Antiretroviral therapy (ART) has made great strides in recent decades, along with the use of preexposure prophylaxis (PrEP), which prevents HIV from replicating in the body [23]. After a decade of hard work, combined antiretroviral therapy (cART), also named highly active antiretroviral therapy (HAART), was introduced for the treatment of this disease. It consists of a cocktail of anti-retroviral drugs. It is the treatment approved to control HIV infection, based on multiple antiretroviral drugs that belong to different classes: NRTIs, NNRTIs, PIs, INSTIs, and entry inhibitors. However, antiretroviral therapy is associated with adverse effects and comorbidities. The appearance of several strains resistant to these drugs represents a real problem that needs to be addressed [24]. 

Moreover, it must be noted that there are situations in which peculiar attention should be paid in the choice of cART, such as during pregnancy, in neonates and in adolescents [25]. Drug development dosing is especially complex for neonates because of their rapidly changing weight, differences in drug absorption and distribution and maturing renal and liver function. As a result, there are very limited treatment options for neonates with HIV infection, and there is an urgent need to address that gap [26]. The management of adolescents living with HIV represents a particular challenge in the global response to HIV. The challenges specific to this age group include difficulties engaging and maintaining them in care, challenges with transition to adult care, and limited therapeutic options for treatment-experienced patients, all of which have been jeopardized by the COVID-19 pandemic [27].

Although the implementation of cART has rendered HIV infection clinically manageable, cART remains a life-long therapy that is not able to eradicate HIV infection. Indeed, the early establishment of long-lived viral reservoirs upon infection and the very limited effectiveness of the current antiviral therapy on the latently infected cells strongly hamper the achievements of HIV cures [28]. In this regard, several studies have shown that HIV is able to persist in blood monocytes and tissue macrophages, even in virologically suppressed people with HIV [29]. HIV DNA has been found in highly purified monocytes and macrophages isolated from the gut, urethra, liver and brain of virologically suppressed people with HIV [30]. In particular, HIV-infected macrophages have been demonstrated to retain an intact and fully competent HIV-1 genome, capable of promoting viral rebound and reseeding the pool of infected cells upon treatment interruption, further reinforcing the challenges posed by the persistence of HIV reservoirs at the level of myeloid cells in terms of HIV cures [28]. Thus, new studies and new therapies that could also target viral reservoirs are urgently needed to curb this disease, increasing the chances to finally achieve a HIV cure. 

In light of this, the main goal for complete HIV-1 eradication is purging latently infected cells from patients’ bodies. A possible strategy called “lock-in apoptosis” targets the budding phase of the lifecycle of the virus, leading to susceptibility to apoptosis of HIV-1-infected cells for the elimination of HIV-1 reservoirs and ultimately for a complete eradication [31]. The so-called ‘shock and kill’ cure approach attempts to activate latent proviruses in HIV-1-infected cells and afterwards kill these cells with strategies including therapeutic vaccines and immune enhancement. This review provides a detailed account of the types of monotherapy and combination therapies against HIV-1 and of the clinical studies currently underway for the treatment of HIV patients, by focusing on the efficacy of the different antiviral approaches on HIV reservoirs, with a peculiar spotlight on MDM, representing the reference model that has been most widely used to evaluate the effectiveness of antiretroviral agents on the myeloid compartment. 

## 2. HIV Reservoirs and cART

To date, it is well established that, although antiretroviral therapy can efficiently suppress viral replication by reducing HIV-RNA to undetectable levels in blood compartments, HIV can persist, even under fully effective cART in specific cellular reservoirs (primarily memory T cells but also MDM, astrocytes and follicular cells). In these cellular reservoirs, HIV can remain under diverse degrees of activity, ranging from latency through viral protein expression up to actively viral replication (mostly at low-level) [32]. Of note, these long-lived reservoir cells, particularly monocyte/macrophages, can resist the cytopathic effects of HIV infection/replication, thus favouring virus dissemination and persistence during infection [33,34]. In particular, several studies have demonstrated that HIV-1 can be detected in circulating monocytes and tissue-resident macrophages from patients on prolonged cART [35,36,37]. Notably, despite these cells not usually being able to produce the infectious virus under basal conditions, they can fuel HIV reactivation following specific stimulation [35,37]. Another key feature of monocyte/macrophages is represented by their capability to mediate HIV spread to CD4+ T cells by fusing with autologous and heterologous CD4+ T cells [38]. Particularly, there is an increasing body of evidence supporting the presence of replication-competent inducible HIV within macrophages isolated from distinct mucosal tissues in the body (such as the gastrointestinal tract, penile urethra, testes and vaginal tissues) or in tissue resident macrophages from different anatomical sites (such as alveolar macrophages in the lungs or microglia and perivascular macrophages in the central nervous system) [39]. In light of this, targeting these cellular sites of infection represents an extremely relevant topic, not only for the achievement of a complete virological suppression under the current ART regimen but also for the future success of functional cure strategies against HIV.

Furthermore, it should also be taken into account that the effectiveness of cART can be suboptimal in some specific HIV target cells such as macrophages in respect to CD4 T cells, thus contributing to HIV persistence in these reservoirs [40,41,42] and challenging the success of both current and novel antiretroviral therapy.

Specifically, according to in vitro experiments, the activity of NRTIs and PIs has been demonstrated to be similar in MDM and in CD4+ lymphocytes [43]. However, markedly increased EC_50_ values for both drug classes have been observed during the chronic infection of macrophages in respect of lymphocytes [44], with relevant clinical implications for the achievement and maintenance of virological suppression in this pivotal cellular HIV reservoirs. Conversely, no substantial differences in the antiviral activity of NNRTIs and INSTIs have been observed between MDM and CD4+ lymphocytes [44], with some studies suggesting that INSTIs could be active even at lower concentrations than those observed in PBMCs [45,46,47].

Data on the antiviral efficacy of entry inhibitors at the level of MDM are more controversial, with some studies indicating that HIV variability and different co-receptor usage could modulate the inhibitory activity of this drug class.

Lastly, information on the efficacy of new antiretroviral drug classes (such as CD4 attachment inhibitors, capsid inhibitors and monoclonal antibodies) is limited or even completely missing. The activity of antiretroviral drugs (classical and upcoming) in MDM will be discussed in more detail in the specific sections dedicated to each single drug class.

This scenario is further complicated by the incomplete and only partially suppressive drug penetration in some anatomical compartments, allowing persistent HIV-1 replication in specific body tissues despite undetectable viremia. 

In particular, it has been demonstrated how the continuous virus production from these sanctuary anatomical sites, protected from cART penetration, can promote viral traffic to blood or lymphoid tissues, even under effective cART, and in turn can replenish the pool of infected cells, being also responsible for viral re-uptake after therapy suspension [48]. In this regard, a recent study, based on advanced molecular techniques applied to autopsies, has highlighted the presence of proviral HIV-DNA in 28 different tissues, including the liver, spleen, genital tract and brain [49]. Accordingly, these tissues represent major sites in which HIV reservoir cells (memory CD4+T cells, dendritic cells, macrophages and microglia) are widely located [43,50].

Overall, these pieces of evidence highlight the barriers to delivering antiretroviral drugs at clinically effective concentrations in all infectious HIV-1 body compartments and to achieving a complete antiviral effect on all HIV target cells, underlining how HIV cellular reservoirs and anatomical HIV sanctuaries represent the major obstacles against the achievement of a functional cure for HIV-1 infection.

## 3. Drug Therapies for HIV Infection

Treating HIV/AIDS is a critical but challenging clinical goal. Before cART introduction, AIDS was the primary killer disease in the US in 1993. After a decade of hard work, cARTs have now been introduced for the treatment of this disease. cART consists of antiviral drug cocktails of anti-retroviral therapies. However, cARTs have their own shortcomings in terms of HIV infection. Nowadays, four decades after the onset of the HIV/AIDS pandemic, the status of the disease has profoundly changed from a near fatal disorder following complications of opportunistic infections to a chronic disease in which renal cardiovascular, diabetes, malignancy, rheumatic and autoimmune disorders have become the most relevant comorbidities to manage [51,52]. In addition, multi-drug resistance (MDR) in HIV has enhanced in recent years. Thus, several efforts and updating therapeutics are under study, which include new generations of drugs, updating HIV vaccines, new types of cART and clinically optimized or even personalized HIV/AIDS therapeutics, both pharmacogenomic and bioinformatic [53,54,55]. Currently, there are more than 30 antiretroviral drugs approved for the treatment of HIV in the US [56,57]. cART effectively silences HIV-1 replication, but the persistence of latent reservoirs in the myeloid [58] and T cells of patients still makes HIV-1 infection incurable [59]. HIV-1 can also replicate in brain microglial cells, which persist despite cART [60]. HIV reverse transcriptase inhibitors are the important components of cARTs for anti-HIV treatment and PrEP in clinical practice. An interesting overview of the historical therapies for the treatment of HIV and other viral diseases has recently been reported by De Clercq (2024) [61]. According to the mechanism of action of antiretroviral drugs, they can be divided into six groups: nucleoside reverse transcriptase inhibitors (NRTIs), non-nucleoside reverse transcriptase inhibitors (NNRTIs), protease inhibitors (PIs), fusion inhibitors (FIs), entry inhibitors (also known as CCR5 antagonist) and integrase strand transfer inhibitors (INSTIs) [62]. Other studies have been applied to recently developed capsid inhibitors such as lenacapavir [63] and monoclonal antibodies such as ibalizumab [64]. The antiviral drugs currently used in therapy for HIV infection are summarized in Table 1. They can be used as either a single regimen or as a combination of three or four drugs [65]. Generally, antiretroviral drugs are used in combination regimens in order to attack multiple stages of the HIV lifecycle, and subsequently slow the disease progression of HIV, thus achieving their highest efficacy, and they are also used to limit the chances of drug-resistant mutants emerging [66]. The most common side effects of these drugs have been recently descripted in detail by Abadie et al. (2023) [67]. 

### 3.1. Nucleoside (Or Nucleotide) Reverse Transcriptase Inhibitors (NRTIs)

NRTIs are drugs able to mimic nucleosides, thus inhibiting HIV reverse transcriptase, slowing the replication of the virus and the progression of the disease. The extensive use of this drugs has led HIV to acquire drug resistance against NRTIs [68]. They include acyclic nucleoside phosphonates (ANPs, such as tenofovir disoproxil fumarate) and nucleoside analogues (lamivudine and tenofovir also being active against HBV). Nucleoside analogues (NUCs) act as chain terminators when their metabolites are incorporated into the viral DNA strands while they undergo replication by polymerases or reverse transcriptase, and they both function similarly with respect to controlling HBV and HIV. ANPs are structurally different from nucleoside analogues, such as lamivudine, in that they have a phosphonate moiety. ANPs have prolonged action [69]. Moreover, it was recently demonstrated that, unlike nucleoside analogues, only ANPs can induce interferon (IFN)-λ3 into the gastrointestinal tract, strongly suggesting that ANPs are not only distinct from nucleoside analogues in their structures but also in their functions [70]. Acyclic nucleoside phosphonates (ANPs) represent a class of viral polymerase inhibitors, designed to mimic natural nucleotides. They are characterized by a non-hydrolysable P–C bond, in which the phosphonate group is linked to the alkyl side chain of purine and pyrimidines [69]. ANPs contain a heterocyclic nucleobase, but the sugar moiety is replaced with an acyclic linker. The introduction of the phosphonate moiety instead of the phosphate one leads to greater stability against in vivo enzymatic hydrolysis [71]. Their discovery originated from the collaboration of Prof. de Clercq with Prof. Antonín Holý, which spanned a period of four decades (1976–2012), and the first (cidofovir, adefovir and tenofovir) were clinically developed by Gilead Sciences [72]. The phosphonate group allows ANPs to interfere with the normal pathway of nucleic acid biosynthesis and, in particular, viral nucleic acid biosynthesis. Unlike “classical” anti-herpes nucleosides, the intracellular activation of ANPs is not dependent on a viral-encoded kinase to promote their initial phosphorylation. Generally, ANPs are divided into diverse subclasses depending on the side chain residue to which a specific antiviral activity spectrum corresponds [73]. Nephrotoxicity is the most common side effect of these compounds [74].

The activity of NRTIs in MDM has been demonstrated by in vitro experiments to be higher than in CD4+ lymphocytes [43]. This is related to the dependence of NRTIs activity on two factors: (i) the intracellular concentration of their triphosphorylated moiety since they require triphosphorylation by cellular kinases to act as competitors of the natural 2′-deoxynucleoside trisphosphates (dNTPs); (ii) the concentration of the cellular dNTP pools. In light of this, since macrophages, as resting cells, are characterized by a limited DNA synthesis and, in turn, a low intracellular dNTPs levels (6- to 20-fold lower than in macrophages) [43,75], in these cells there is a lower competition with natural dNTPs for triphosphorylation by cellular kinases than in CD4+ lymphocytes. Overall, this can explain the higher NRTIs efficacy in MDM. However, during chronic HIV-1 infection, it is known that continuous exposure to the macrophage colony-stimulating factor might activate macrophages, resulting in increased dNTP levels. In turn, this can determine an increased competition for the cellular kinases and, thus, can decrease the ability of the NRTIs to be functionally active. Together, these mechanisms might be responsible for the markedly increased EC_50_ values for NRTIs observed in the setting of chronic infection in macrophages in respect to lymphocytes [44].

#### 3.1.1. Zidovudine or Azidothymidine

The first antiretroviral drug introduced for therapy was zidovudine (3′-azido-2′,3′-dideoxythymidine, ZDV, AZT, also known as azidothymidine, Retrovir^®^), and was the analogue of thymidine, first synthesized in 1964, and licensed by the US FDA in 1987 for HIV treatment [76]. This drug was initially developed as an anticancer agent in the 1960s and was thus the first example of ‘drug repositioning’ or ‘drug repurposing’ in the history of medicinal chemistry [77]. AZT is a prodrug: the active form is formed upon triphosphorylation at the 5′-OH position in the cell by kinases. Several studies suggest that AZT can partially reverse HIV-associated neurological disorders, including dementia and peripheral neuropathy. However, these effects seem to be limited and decrease with the prolongation of therapy. In addition, AZT has also been found to be active in blocking T-leukemia virus-1 (human lymphoma) and other mammalian retroviruses [78]. The main adverse effects observed are nausea/vomiting, diarrhoea, headache and bone marrow suppression [79].

#### 3.1.2. Tenofovir, Tenofovir Disoproxil Fumarate and Tenofovir Alafenamide

Tenofovir (TFV), originally named (*R*)-PMPA, was first described in 1993. Once within cells, tenofovir is phosphorylated to tenofovir phosphate (TFVp), and then to tenofovir diphosphate (TFVpp), a structural analogue of deoxyadenosine 5′-triphosphate (dATP) that inhibits the elongation of viral DNA by targeting the RNA-dependent DNA polymerase (reverse transcriptase) of either HIV or HBV [80]. Two orally bioavailable prodrug forms, specifically tenofovir disoproxil fumarate (TDF, Viread^®^) [81] and tenofovir alafenamide (TAF, GS-7340, Vemlidy^®^) [82] were then described. TDF and TAF are both available for the treatment of hepatitis B virus (HBV) infections and, in combination with emtricitabine, for the prophylaxis of HIV infections. 

TDF is the first-line antiviral therapy for chronic viral hepatitis B. It is an orally bioavailable prodrug that is rapidly metabolized to the active component tenofovir in plasma. However, long-term use has led to renal failure and hypophosphatemic osteomalacia. The impact of TDF antiretroviral regimens is mostly in regard to changes in bone health [83]. Secondary hyperparathyroidism and renal Fanconi syndrome have also been reported [84]. 

TAF can be considered the successor of TDF as a prodrug of tenofovir [85]. The FDA approved the combination TAF/emtricitabine for PrEP in 2019 for non-vaginal sex [86]. TAF is specifically accumulated in lymphatic tissue, and in the liver, and hence also holds great potential for the treatment of hepatitis B virus (HBV) infections [87]. Numerous clinical trials have consistently demonstrated the significant lesser impact of TAF vs. TDF on both renal function and bone mineral density [88,89]. Interestingly, a recent randomized study by Soldado-Folgado also showed that an improvement in bone quality was found after switching from a TDF- to a TAF-based antiretroviral therapy, independent of bone mineral density [90].

#### 3.1.3. Lamivudine 

Lamivudine (2′-deoxy-3′-thiacytidine, 3TC or LAM, Epivir^®^, Zeffix^®^) is an analogue of cytosine that potently affects HIV replication by inhibiting viral reverse transcriptase enzymes, acting as a DNA chain terminator. It is a chiral drug, specifically the L-form, as it revealed a higher activity than the natural enantiomer (D-form). The L-enantiomer is markedly less cytotoxic in human lymphocyte cultures than its isomer; thus, it was developed as an antiretroviral drug. It was approved in 1995 for the treatment of HIV-1 infection and in 1998 for hepatitis B virus (HBV) infection. After approximately 30 years, lamivudine remains a well-established component in the diverse treatments used for HIV infection and emerging therapies, even in recent innovative treatment strategies, such as single-tablet regimens containing potent 3- or 4-drug combinations [91]. Combivir^®^, which is the combination of zidovudine and lamivudine, was approved by the FDA in 1997 [92]. Given the excellent efficacy and safety profile and the availability of low-cost generic versions, lamivudine is commonly used in low- and middle-income countries for the treatment of HIV infection. In addition, it has a well-established pharmakinetic profile with few drug–drug interactions, which is important not only for the convenience of combining 3TC with other drugs in single-tablet regimens but also because of the prevalence of multiple comedications in certain populations, such as the elderly or hepatitis-coinfected patients. However, recent studies are underway to assess the environmental impacts of lamivudine as an emerging contaminant, at different trophic levels, to both flora and fauna, at concentrations previously found in the environment [93,94,95].

#### 3.1.4. Emtricitabine

Emtricitabine (FTC) is a synthetic cytidine nucleoside analogue that received approval from the FDA on 2 July 2003, under the trade name Emtriva^®^ (formerly Coviracil^®^), for HIV therapy once a day in adults. Its structure differs from lamivudine only due to the presence of a fluorine atome at the 5-position of the cytosine ring [96]. It is phosphorylated to emtricitabine 5′-triphosphate which, in turn, inhibits the activity of HIV-1 reverse transcriptase by competing with the endogenous substrate deoxycytidine 5′-triphosphate for binding to HIV-1 reverse transcriptase [97]. After its first approval, several combinations with other antivirals have been introduced onto the market for the prevention and/or treatment of HIV [98]. 

#### 3.1.5. Didanosine 

Didanosine (2′,3′-dideoxyinosine, ddI) is a synthetic inosine/adenosine/guanosine analogue and highly active antiretroviral therapeutic agent used in combination with antiretroviral regimens for the treatment of human immunodeficiency virus infection and acquired immunodeficiency syndrome (HIV/AIDS). It was introduced in 1991 with the trade name Videx™. It was removed from the market due to adverse effects [99], including retinal toxicity in children [100] and adults [101], and drug interactions. This potent reverse-transcriptase inhibitor has recently been reconsidered as a repositioned drug and is characterized by proven strong pharmacological effects against the viral genome, and may therefore successfully take part in the effective treatment of COVID-19 [102].

#### 3.1.6. Stavudine-d4T

Stavudine (d4T, Zerit^®^) was approved by the US FDA in 1994 for HIV treatment. However, its use could result in HIV-associated sensory neuropathy (HIV-SN) and lipodystrophy [103]. Since stavudine was removed from recommended treatment schedules, the prevalence of HIV-SN has declined; thus, stavudine has not been recommended since 2010 and is now rarely prescribed [104]. However, recent studies are underway to understand if there really is a clear relationship between these two pieces of evidence, also in relation to polymorphism [105,106].

#### 3.1.7. Abacavir

Abacavir (ABC, Ziagen^®^ containing abacavir sulfate) is an NRTI approved by the US FDA in December 1998 [107]. It is a carbocyclic 2′-deoxyguanosine nucleoside analogue, which is metabolised intracellularly to a 2′-deoxyguanosine nucleoside analogue which competitively inhibits HIV reverse transcriptase and terminates proviral DNA chain extension [108]. Its side effects are related to the cardiovascular system: abacavir-based therapies may increase the risk of cardiovascular diseases compared with abacavir-free regimens [109,110]. Several studies are currently underway to understand the mechanisms responsible for this effect. It has been related to the neutrophil P2X7 receptor and low-density lipoprotein receptor-1 (LOX-1), as reported by Blanc-Ruiz [111], and it is likely that abacavir affects the kinetics of prothrombin conversion rather than procoagulant factor levels, as shown by Yan et al. [112]. Moreover, hypersensitivity reactions have been observed [113,114]. It has been used in clinical studies in combination with dolutegravir and lamivudine in dispersible and immediate-release tablets for children with HIV (IMPAACT 2019, phase 1–2 study) [115] and in paediatric abacavir–lamivudine fixed-dose dispersible tablets and ritonavir-boosted lopinavir granules for neonates exposed to HIV (PETITE, phase 1–2 study) [116]. 

#### 3.1.8. Islatravir

Islatravir (ISL, MK-8591) is a nucleoside reverse transcriptase translocation inhibitor (NRTTI) [117]. It acts with a different mechanism from other NRTIs. It inhibits HIV RT through multiple mechanisms, such as defects in the translocation of RT along the template-primer. It is an ultrapotent antiviral with high tolerability, as demonstrated by in vitro and clinical studies [118]. However, in vitro studies have revealed the emergence of resistance mutations and their prevalence as natural polymorphisms. It has been suggested that a complementary profile with doravirine may result in a two-drug regimen (NCT03272347).

### 3.2. Non-Nucleoside Reverse Transcriptase Inhibitors (NNRTIs)

NNRTIs do not act as chain terminators; they bind to a hydrophobic pocket in the reverse transcriptase enzyme, leading to the direct inhibition of HIV-1 RT function. Their antiviral effect it is not affected by the deoxyribonucleoside triphosphate pool [44]. NNRTIs include first-generation inhibitors, nevirapine and efavirenz, and second-generation inhibitors, etravirine, rilpivirine, doravirine and dapivirine, which is only used in vaginal rings to prevent HIV-1 infection.

Considering that the activity of NNRTIs is not affected by the size of dNTP pools as described for NRTIs, substantial differences in the antiviral activity of NNRTIs have not been observed between MDM and CD4+ lymphocytes [44]. Accordingly, the anti-HIV-1 activity of NNRTIs is not altered by the macrophage colony-stimulating factor, which is known to enhance the dNTPs pool in MDM and is thus capable to affect the full activity of NRTIs [42].

#### 3.2.1. Nevirapine

Nevirapine (NVP, Viramune^®^) was the first approved NNRTI to target HIV. It received approval in 1996 and is still broadly used in developing countries, also in neonates, for combinatorial antiretroviral and prophylactic therapies against HIV infection, but it can cause idiosyncratic drug reactions with severe skin rashes and hepatotoxicity [119].

#### 3.2.2. Efavirenz 

Efavirenz (EFV, Sustiva^®^, Stocrin^®^) was approved by the FDA in September 1998. Its combination with two nucleoside analogue reverse transcriptase inhibitors is recommended as a preferred first-line regimen for the treatment of HIV-1 infection. Its long plasma half-life enables once-daily dosing, but, as a consequence of this and the low genetic barrier, it is also prone to viral resistance when adherence to therapy is suboptimal. The most common adverse effects are neuropsychiatric symptoms. They are generally transient but have been shown to persist for up to 2 years after initiation of therapy in some patients [120]. Studies in vivo demonstrated that it is able to interact off-target with the CNS-specific enzyme CYP46A1 and allosterically activates this enzyme at a low dose in the brain and retina of mice. Treatment with low-dose efavirenz has recently been suggested as a potential new therapeutic approach for reducing glaucoma risk factors based on CYP46A1 activation [121]. It is also a moderate CYP3A4 inducer [122].

#### 3.2.3. Etravirine 

Etravirine (ETR, Intelence^®^, Tibotec^®^) is a second-generation NNRTI which was approved by the US FDA in January 2008 for the treatment of HIV-1 infection in combination with other antiretroviral agents for treatment-experienced patients who have HIV-1 strains that are resistant to other NNRTIs [123]. It is a potent inhibitor of HIV reverse transcriptase against wild-type and most NNRTI-resistant HIV strains. It can be administered twice-daily, although once-daily dosing is being investigated in treatment-naïve and treatment-experienced persons. Etravirine is used as a third- or fourth-line antiretroviral therapy. Etravirine is metabolized extensively through CYP450 enzymes exhibiting inhibitory (CYP2C19, CYP2C19) and inductive (CYP3A4) properties, posing various considerations for drug–drug interactions [124].

#### 3.2.4. Rilpivirine 

Rilpivirine (RPV, Edurant^®^, containing rilpivirine hydrochloride) was approved in 2011. It inhibits HIV-1 replication via non-competitive inhibition of viral reverse transcriptase, thus preventing the synthesis of viral DNA and hence subsequent integration of the viral genome into host DNA [125]. It has demonstrated aggregation-induced emission [126]. Recently, the usefulness of rilpivirine has been demonstrated in the management of thrombotic cardiovascular disease (CVD), which usually affects HIV-infected individuals. Rilpivirine is able to prevent the progression of thrombotic cardiovascular diseases by interrupting β3-integrin-mediated outside-in signalling via inhibiting c-Src activation without haemorrhagic side effects, and it is thus being suggested for the prevention and therapy of these diseases [127].

#### 3.2.5. Doravirine

Doravirine (DOR, Pifeltro™) was approved by the US FDA on 30 August 2018, for the treatment of HIV infection in adult patients. It then received approval in the EU and Japan in November 2018 and January 2020, respectively. It is currently available as a single stand-alone tablet as well as part of a single-tablet regimen in a fixed-dose combination with tenofovir disoproxil and lamivudine. Doravirine has a more favourable drug interaction profile compared with earlier NNRTIs as it neither inhibits nor induces the cytochrome P450 3A4 (CYP3A4) enzyme. Doravirine has been added to the category of Recommended Initial Regimens in Certain Clinical Situations in the United States Department of Health and Human Services Antiretroviral Guidelines for Adults and Adolescents [128].

#### 3.2.6. Dapivirine

Dapivirine (DPV) is a discreet anti-HIV microbicide created to be used monthly, specifically for women as a vaginal ring. In July 2021, the WHO released updated consolidated guidelines that included a recommendation for dapivirine as a prevention choice for individuals at substantial risk of HIV infection [129]. It has since received a positive scientific verdict by the European Medicines Agency and is included in the WHO HIV prevention guidelines [130]. It is safe to use and effective at reducing women’s risk of acquiring HIV infection. It is currently not recommended for use during breastfeeding. However, clinical studies are being carried out for the use of dapivirine vaginal rings and oral daily PrEP among breastfeeding persons (Microbicide Trials Network 043/B-PROTECTED, a phase 3B study) [131,132].

### 3.3. Protease Inhibitors (PIs)

Protease inhibitors have been traditionally developed by natural product screening for lead compounds with subsequent optimization or by empirical substrate-based methods [133]. In the lifecycle of HIV, protease is an essential element for viral maturation. Some of the most potent antiretroviral agents belong to this class and inhibit HIV-1 protease activity by blocking its active site [134]. However, their rapid metabolism in the intestines and liver, predominantly by cytochrome P450 (CYP) 3A, can lead to low systemic exposure. Co-administration of a pharmacokinetic enhancer has greatly contributed to the effective and safe therapeutic profile of PIs. However, poor bioavailability and toxicity are their common disadvantages. Drug resistance is a major problem in HIV-1 protease-inhibitor-based chemotherapy, which is characteristically associated with the decreased binding affinity of the inhibitor with the protease.

All clinically available PIs showed antiviral activity in chronically infected MDM. Nevertheless, PI activity occurs at EC_50_ values greater than those required in chronically infected CD4+ T lymphocytes [135,136,137,138]. The lower PI activity in MDM versus CD4+ lymphocytes may be imputable to the high RNA metabolism in these cells, sustaining a huge production of viral particles, even from a limited amount of proviral HIV-DNA. This evidence may have relevant clinical consequences. Indeed, the high concentration of PIs required to suppress HIV-1 replication in chronically infected MDM is often beyond the PI concentration achievable in the plasma of treated patients. In light of this, tissue macrophages may escape HIV suppression, particularly in patients with a suboptimal adherence to therapy or an altered drug absorption or metabolism.

#### 3.3.1. Saquinavir 

Saquinavir (SQV, Invirase^®^, developed by F. Hoffmann-La Roche Ltd., Basel, Switzerland), received its approval in 1995. It was the first FDA-approved HIV protease inhibitor used in the treatment of patients with AIDS. However, saquinavir is not a preferred protease inhibitor regimen due to its low bioavailability, probably associated with a mix of elevated first-pass metabolism and incomplete absorption [139]. The repositioning of this drug has been suggested for the treatment of COVID-19, *Mycobacterium tuberculosis* infections and cancer diseases, including sarcoma, neuroblastoma, cervical cancer and leukaemia [140,141]. Drug-resistant mechanisms related to saquinavir are currently under study [142].

#### 3.3.2. Ritonavir

Ritonavir (RTV, RIT, Norvir^®^, developed by Abbott Laboratories, Abbott Park, IL, USA) was approved by the FDA in 1996. It was originally designed as an HIV protease inhibitor, but it was found later that it also inhibits CYP3A4, boosting the circulating concentration of other HIV protease inhibitors and preventing the metabolism of other protease inhibitors. However, boosting with ritonavir shows some limitations, such as increased pill burden, adverse effects and a wide range of clinically significant drug–drug interactions. CYP3A4 inhibition is not selective, and ritonavir may also induce the activity of enzymes such as CYP1A2, CYP2C9, CYP2C8, CYP2B6, CYP2D6 and CYP2C19, as well as P-gp and glucuronyltransferases, resulting in numerous off-target drug interactions [143,144]. Several reports suggest a positive association of hyperlipidemia with the use of PI (including ritonavir)-based regimens [145], along with other harmful effects such as hepatotoxicity, cardiovascular diseases and testicular oxidative damage [146,147]. Ritonavir has been recently used for COVID-19 treatment (e.g., Paxlovid). Its side effects are well manageable, even in chronic administration regimens, and thus it is still the clinically most-used pharmacokinetic enhancer [148].

#### 3.3.3. Indinavir

Indinavir (IDV, Crixivan^®^, developed by Merck & Co, Inc., Whitehouse Station, NJ, USA) was approved in 1996. It effectively inhibits both HIV-1 and HIV-2; however, the concentration of circulating indinavir decreases rapidly, often leading to treatment failures. Moreover, its low solubility may lead to the development of kidney stones. In addition, indinavir can behave as a competitive inhibitor of the cytoplasmic glucose binding site of glucose transporter GLUT4 [134]. Further, indinavir has a short acting time and requires a dosage of 800 mg every 8 h. For these reasons, indinavir has been replaced by second-generation protease inhibitors. 

#### 3.3.4. Nelfinavir 

Nelfinavir (NFV, Viracept^®^ containing nelfinavir mesylate) was approved in 1997 in combination therapy for the treatment of HIV infection. The recent COVID-19 pandemic [149,150] has brought it back to the limelight. Nelfinavir markedly improved lung pathology in SARS-CoV-2-infected Syrian hamsters, despite the lack of an antiviral effect [151]. Moreover, the activity of nelfinavir in cancer has been widely reported. Phase 1 and 2 clinical trials have proven the safety, tolerability and positive outcome of nelfinavir in cancer patients, with or without co-treatments against pancreatic cancer, multiple myeloma, non-small-cell lung carcinoma and locally advanced cervical cancer [152,153,154,155].

#### 3.3.5. Amprenavir

Amprenavir (APV, Agenerase^®^) received FDA approval in 1999 [98]. It has been recently found that amprenavir inhibits pepsin, and the antireflux therapeutic potential of its prodrug fosamprenavir has been found in a mouse model of laryngopharyngeal reflux [156]. The protection against pepsin-induced cell dissociation, E-cadherin cleavage and MMP induction of amprenavir has suggested a potential therapeutic role for amprenavir in gastroesophageal reflux disease (GERD), recalcitrant to proton pump inhibitor therapy, and for preventing GERD-associated neoplastic changes [157].

#### 3.3.6. Fosamprenavir

Fosamprenavir (FPV) is the prodrug of amprenavir. Through metabolic processes within the human body, fosamprenavir undergoes biotransformation to form amprenavir, which represents the active moiety responsible for therapeutic effects; thus, it may be considered a slow-release formulation of amprenavir. Fosamprenavir was first approved by the US FDA on 20 October 2003, and by the EMA on 12 July 2004, under the trade names Lexiva^®^ (in the US.) and Telzir^®^ (in Europe) and contains fosamprenavir calcium hydrate [95]. It is mainly used with NRTI for the treatment of HIV-infected patients. In Japan, Lexiva tablets (fosamprenavir calcium hydrate) have been marketed since January 2005 and used in clinical practice. It generally has a good safety profile [158].

#### 3.3.7. Lopinavir 

Lopinavir (LPV) has been commercialized in combination with ritonavir with the name Kaletra^®^ and contains 33 mg ritonvir and 133 mg LPV for the treatment of HIV infection. It received approval by the FDA in 2000. The antiviral effect is due to LPV, but it is inactivated by CYP3A; thus, it has been combined with ritonavir, which acts as a ‘booster’ by virtue of its inhibitory effect on the enzyme [159]. The combination lopinavir/ritonavir was used in the first stages of the COVID-19 pandemic [160,161], but it was then found that this combination did not improve outcomes relative to placebo for COVID-19 [162].

#### 3.3.8. Atazanavir

Atazanavir (BMS-232632, ATZ, ATV, Reyataz^®^) received approval from the FDA on 20 June 2003. It prevents the formation of mature virions in HIV-1-infected cells by inhibiting the cleavage of *gag* and *gag-pol* polyproteins. It is administered once daily, thus alleviating the need for multiple daily administrations. Moreover, it exhibits a more favourable impact on the lipid profile of patients in comparison with other PIs, resulting in less pronounced alterations in blood cholesterol and lipid levels. It is used exclusively in combination with other medications targeting HIV [163].

#### 3.3.9. Tipranavir

Tipranavir (TPV, Aptivus^®^) is a nonpeptidic PI developed by Boehringer Ingelheim that received regulatory approval from the FDA on 22 June 2005. Then, on 24 June 2008, it was granted authorization for paediatric use [164]. It is utilized in combination with ritonavir for the management of HIV infection. Tipranavir is able to effectively inhibit viral replication in cases where resistance to other PIs was observed; thus, it represents a viable treatment option for patients who have developed resistance to alternative therapeutic approaches. Tipranavir should be administered only in conjunction with ritonavir and additional antiretroviral drugs, and it does not possess approval for usage in treatment-naïve patients. The side effects of tipranavir include gastrointestinal reactions, hepatotoxicity, hypercolesterlemia and hypertriglyceridemia [165].

#### 3.3.10. Darunavir 

Darunavir (DRV) belongs to the new generation of PIs and shows a great advantage in the treatment of HIV/AIDS given its superior efficacy on wild-type HIV and on already resistant strains. The compound was licensed for marketing in the US in 2006, and in 2007 in Europe as darunavir ethanolate (Prezista^®^). Since 2016, DRV has been a treatment option for children over three years of age, adolescents and naive adults infected with HIV-1, but also for critical patients [166]. It is frequently recommended along with low doses of enzyme inhibitors such as cobicistat and ritonavir. Each Rezolsta^®^ film-coated tablet contains 800 mg of darunavir ethanolate and 150 mg of cobicistat [167]. Boosted darunovir is well tolerated by patients, with fewer side effects than other regimens.

### 3.4. Integrase Strand-Transfer Inhibitors (INSTIs)

INSTIs represent an attractive class of next-generation antiretroviral drugs that have gained attention in the last 15 years [168]. Since 2007, five INSTIs have been introduced: raltegravir, elvitegravir, dolutegravir, bictegravir and cabotegravir [169,170]. They bear a polycyclic core with heteroatom triads and a halogenated benzyl group that interacts with the vcDNA attached to the CCD by a flexible linker [171]. These drugs inhibit the strand transfer step of viral DNA integration into the host chromosome [172]. INSTIs are a generally well-tolerated class of antiretrovirals and have a higher antiviral potency compared to other classes of antiretroviral agents [173]. A computational study has recently beencarried out to design new INSTIs against emerging and evolving drug-resistant HIV-1 integrase mutants [174]. One limitation of INSTIs is potential cross-resistance due to mutations Q148H and R263K, which is still lower compared with other antiretroviral agents [175].

Regarding the activity of INSTIs on MDM, several studies have demonstrated their activity in inhibiting HIV in these cells at similar, or even lower, concentrations than those observed in PBMCs [45,46,47]. Furthermore, in vitro studies have confirmed that second-generation INSTIs also retain antiviral activity against raltegravir-resistant HIV variants (carrying the Y143 or N155 resistance pathways) at comparable potency in both MDM and CD4+ T cells.

#### 3.4.1. Raltegravir 

Raltegravir (RAL, Isentress^®^) was the first INSTI approved by the US FDA, in October 2007, for the treatment of HIV-1 as part of combination antiretroviral therapy in treatment-experienced patients, providing an additional option for the management of HIV-1-infected individuals [176]. It targets the strand transfer step of HIV-1 integration [177]. It has recently been implicated as a promising drug in cancer treatment; thus, it has been recently suggested as a potential repositioned drug for the treatment of multiple myeloma [178]. In addition, in vitro and in vivo studies have demonstrated its efficacy in inhibiting human colorectal tumour cell invasion and metastasis in a Fascin1-dependent manner [179]. In combination with lamivudine, it has shown high levels of ex vivo HIV protection PrEP, with high drug concentrations persisting after discontinuation in vaginal and rectal compartments [180]. This combination is suggested as a maintenance regimen in virologically suppressed persons with HIV [181].

#### 3.4.2. Elvitegravir 

Elvitegravir (EVG, Vitekta^®^) is the second INSTI to be approved by the US FDA. It received approval in 2012 as a fixed-dose combination with cobicistat (a pharmacokinetic booster) and emtricitabine and TDF as an initial therapy for treatment-naive patients with HIV/AIDS (FDA, 2012), and in 2014 as an individual drug to be used in combination with ritonavir-boosted protease inhibitors plus other antiretrovirals for treatment-experienced HIV-positive adults, although this has since been discontinued by the manufacturer [182].

#### 3.4.3. Dolutegravir

Dolutegravir (DTG, Tivicay^®^) received approval by the FDA and European Medicines Agency (EMA) in 2013 and 2014, respectively, for the treatment of HIV-1 infection in combination with other antiretroviral agents in adults and adolescent children (more than 6 years) [183]. It is used in first-line and second-line antiretroviral therapy. Along with tenofovir and lamivudine, it is the WHO-recommended first-line regimen [184]. It has potential advantages in comparison to first-generation INSTIs, including unboosted daily dosing, limited cross resistance with raltegravir and elvitegravir and a high barrier to resistance [185,186,187]. Several clinical studies have been carried out with this medication, as detailed below. 

#### 3.4.4. Bictegravir

Bictegravir (BIC) is a second-generation ISTI which was approved by the FDA and EMA in 2018 as part of a single-tablet regimen developed by Gilead Sciences (Biktarvy^®^—a fixed-dose once-daily triple-drug regimen of bictegravir, emtricitabine and tenofovir alafenamide) [188,189]. It shows high resilience to INSTI-resistant mutations [190]. This combination is recommended as initial and long-term therapy for the treatment of HIV infection. For PrEP, TDF or TAF remains recommended, but bictegravir may possibly be added [191]. 

#### 3.4.5. Cabotegravir 

Cabotegravir (CAB) was approved by the FDA as tablets (Vocabria^TM^), which should be taken in combination with oral rilpivirine (Edurant^®^) [192]. Cabenuva^®^, which contains cabotegravir extended-release injectable suspension and rilpivirine extended-release injectable suspension, was the first injectable antiretroviral drug approved by the US FDA, in January 2021, for use in treating HIV in adults [193]. Cabenuva^®^ was designated “Fast Track” and “Priority Review”, which served to speed along the drug to market with special attention from drug developers. Apretude^®^, which was an analogue of Cabenuva^®^, was approved by the FDA on 20 December 2021, for use in preventing HIV in at-risk youth and adolescents. It represents the first injectable PreP medication [194]. Cabotegravir ER is slow to be absorbed and has a long elimination half-life, both of which permit infrequent dosing (1 month apart for two consecutive months, and every 2 months thereafter). It is generally well tolerated. Cabotegravir ER injectable suspension is indicated in the USA for PrEP to reduce the risk of sexually acquired HIV-1 infection in at-risk adults and adolescents weighing ≥35 kg who have a negative HIV-1 test prior to initiation. In clinical trials, cabotegravir ER injectable suspension showed higher efficacy than oral daily emtricitabine/tenofovir disoproxil fumarate in preventing the acquisition of HIV-1 in at-risk transgender women, cisgender men who have sex with men and cisgender women [195].

### 3.5. Human Liver Cytochrome P450 (CYP) Enzyme Inhibitors/Pharmacokinetic Enhancers

Drugs used to manage HIV infection, especially drugs included in the PI, NNRTI and the INSTI classes, are metabolized via the cytochrome P450 (CYP) pathway. All the PIs are metabolized by CYP3A4, and almost all the PIs are inhibitors of CYP3A4. Other than PIs, efavirenz (EFV) and etravirine (ETR) from the NNRTI class also have the potential to inhibit CYP enzymes. EFV is metabolized by CYP2B6 but can inhibit CYP3A4. ETR is metabolized by CYP3A4, CYP2C9 and CYP2C19, and it is also an inhibitor of CYP2C9 and CYP2C19. To minimize the first pass metabolism, pharmacokinetic enhancers (PK enhancers), ritonavir (RTV) and cobicistat (COBI) are used in ART regimens due to their ability to inhibit CYP3A4, leading to decreased doses and frequency of administration for ARVs which are substrates of CYP3A4. Table 1 provides a summary of the CYP substrates, inhibitors and inducers used in HIV treatments [196]. Cobicistat (COBI, Tybost^®^; Gilead Sciences, Forest City, CA, USA) is a selective CYP3A inhibitor without intrinsic anti-HIV activity. It was approved in 2015 in the EU for HIV, in combination with other antiretroviral agents, as a pharmacokinetic enhancer (i.e., booster) of the HIV-1 PIs atazanavir and darunavir in adults [197,198]. It is present in several combinations, as reported below.

### 3.6. Blocking Viruses Entry Cells

Class I fusion protein envelope glycoprotein (Env) is expressed on HIV surfaces and attacks CD4+ T cells, macrophages and monocytes. It strengthens the attachment of viral particles to target cells and the fusion of viral envelope and target cell membranes. It includes three identical glycoprotein precursors, gp160, and is split into two subunits, the surface glycoprotein gp120 (SU) and the transmembrane structural domain gp41 (TM), during transit to the cell surface. HIV-1 enters target cells by binding its envelope glycoprotein gp120 to the CD4 receptor and/or co-receptors such as C-C chemokine receptor type 5 (CCR5; R5) and C-X-C chemokine receptor type 4 (CXCR4; X4), and R5-tropic viruses predominate during the early stages of infection. During HIV infection, the interaction of the gp120 receptor binding domain with the CD4 cell receptor promotes a sequence of conformational changes in the envelope protein, leading to the dissociation of gp120 from gp41 and the promotion of membrane fusion. HIV entrance inhibitors are broadly categorized into three groups: fusion inhibitors (or entry inhibitors), targeting gp120 and gp41, CD4 attachment inhibitors and chemokine receptor inhibitors [199]. 

#### 3.6.1. Entry Inhibitor (EI) or Fusion Inhibitors

**Figure 1:**
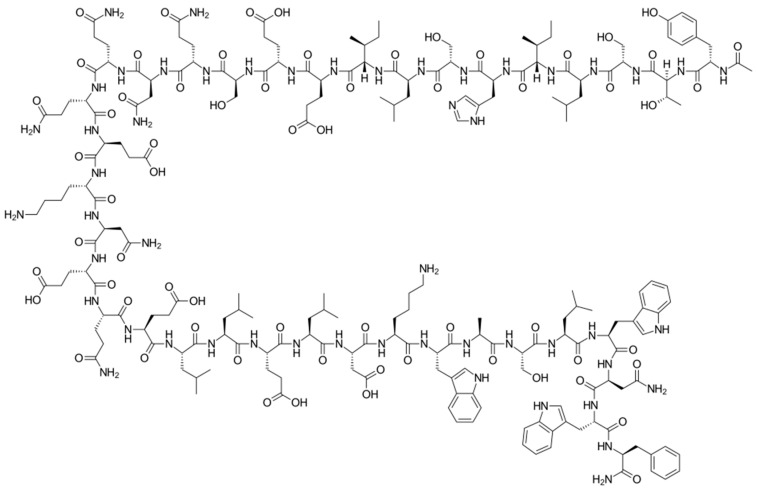
Structure of enfuvirtide.

Enfuvirtide (ENF, T20, Fuzeon^®^) was the first HIV entry inhibitor to receive authorization by the FDA, in March 2003 [200]. It is a synthetic 36-amino acid peptide (Figure 1) and is administered by subcutaneous injection twice daily [201]. 

It demonstrates high antiviral activity, but its application is largely confined by its side effects. Due to the rapid metabolism by peptidase, twice daily administration is required with a dosage of 90 mg per injection, which considerably increases injection site reactions in patients over a long period of treatment [202]. Albuvirtide (ABT, Aikening^®^) was the first long-acting HIV fusion inhibitor, developed and approved in China in 2018, that blocks the invasion of the HIV-1 virus into target cells. This was the first approval of a second-generation, long-acting peptide HIV fusion inhibitor [203]. It irreversibly binds to serum albumin and has a long half-life and potent anti-HIV activity in vivo. ABT can establish a stable, α-helical conformation with the target sequence and inhibit the formation of a fused active hexa-helix bundle, thus preventing HIV-1 Env-mediated membrane fusion and viral entry [204]. A single-centre, randomized, open-label, single-period, parallel phase I clinical trial showed that adverse events were mild, according to the Common Terminology Criteria for Adverse Events (CTCAE 5.0) guidelines and the study investigator’s judgement [205]. It is particularly effective against HIV-1 strains resistant to enfuvirtide [204].

Enfuvirtide has also been demonstrated to efficiently prevent HIV entry into MDM by in vitro experiments. Nevertheless, it is still debated if susceptibility to enfuvirtide can be potentially modulated by the specificity of the co-receptor usage [206]. In particular, early studies suggested that sensitivity to enfuvirtide could be greater for HIV strains using CXCR4 than for those using CCR5, although later reports did not confirm this clear dichotomy [207]. Alternatively, experiments on dual-tropic R5/X4 HIV strains in primary macrophages (known to support the entry of R5/X4 variants through both co-receptors), have shown that sensitivity to enfuvirtide varied among different HIV strains but independently from different co-receptor usage [208], rising concerns on the full effectiveness for all HIV strains in these cellular reservoirs. 

#### 3.6.2. CCR5 Antagonists 

CCR5 antagonists bind to CCR5 to prevent viral entry. The first oral agents belonging to this class were aplaviroc (GlaxoSmithKline, Brentford, UK), maraviroc (Pfizer, Tadworth, UK) and vicriviroc (Schering-Plough Company, Kenilworth, NJ, USA). However, aplaviroc and vicriviroc showed hepatotoxicity and carcinogenicty, respectively, in clinical trials; thus, they were discontinued. Maraviroc (MVC, Selzentry^®^) was the only US FDA drug approved for marketing. It was also approved by the European Commission, Health Canada and several other countries for the treatment of patients infected with R5-tropic HIV-1. Maraviroc represents a special case, as it only inhibits R5 viruses and is ineffective against X4 viruses. Thus, clinical testing of maraviroc resistance (i.e., co-receptor tropism) is needed before initiating maraviroc treatment in patients infected with HIV-1 [209]. Recent studies have demonstrated its activity in various types of cancers and inflammations [210]. Other compounds belonging to this class are under study for HIV infection, such as PRO10 and cenicriviroc [211,212].

CCR5 antagonists, such as maraviroc, have been shown to be effective in vitro against HIV with pure R5 or R5/X4 dual tropism in both MDM and lymphocytes, despite the potential emergence of X4-mediated escape, which can hamper their effectiveness [213]. Indeed, the failure of CCR5 antagonists can result more commonly from the selection of pure CXCR4-using HIV strains under therapy, but also from the development of adaptive gp120 mutations that enable recognition and entry via the drug-bound conformation of CCR5 [214,215,216]. In this latter scenario, it has been demonstrated that the resistant mutations that emerged under maraviroc can dramatically attenuate HIV infectivity, cell–cell fusion activity and viral replication capacity in MDM. In light of this, there could be a beneficial impact of continuing therapy with co-receptor antagonists, in some specific cases, even after the development of resistance [217,218]. Moreover, CCR5-using HIV strains induce a lower percentage of macrophage death compared to CXCR4-using strains and up-regulate macrophages homeostasis, showing the relevance of blocking HIV CCR5 usage in such pivotal cell reservoirs [219]. This is an issue that still deserves further investigation. Overall, to date, the emergence of resistant strains, and particularly the switch to non-R5 tropic HIV variants, has limited the current usage of this drug.

#### 3.6.3. CD4 Attachment Inhibitors

Fostemsavir (Rukobia^®^) is the prodrug of the HIV-1 attachment inhibitor temsavir, a gp120-binding inhibitor that was approved in July 2020 in the USA for use in combination with other antiretroviral agents for the treatment of HIV-1 infection in heavily treatment-experienced adults [220,221]. Temsavir decreases gp120-mediated immunomodulatory activity, including the elimination of antibody-dependent cytotoxicity in uninfected bystander CD4+ T cells and gp120-induced cytokine bursts in peripheral blood mononuclear cells, suggesting that it may provide clinical benefits beyond blocking viral neutralization, particularly by preventing elimination of uninfected cells and induction of a cytokine burst caused by its soluble viral glycoprotein [222]. Temsavir is mainly metabolized through hydrolysis but also via CYP450 oxidation; therefore, it should not be taken with strong CYP3A inducers such as rifampin, carbamazepine, phenytoin, mitotane, enzalutamide or St John’s wort. The most common side effects comprise nausea, diarrhoea, headache, abdominal pain, dyspepsia, fatigue, rash and sleep disturbance [223]. To date, information on the efficacy of fostemsavir in macrophages is largely missing.

## 4. Therapies for Multidrug-Resistant (MDR) HIV Patients

Approximately 25,000 patients living with HIV in the United States are considered to harbour MDR strains, including an estimated 12,000 patients in vital need of new treatment options due to previously failed regimens [224,225]. The prevalence of HIV resistance in adults initiating ART from 2014 to 2020 was higher in the Americas than in Africa, Southeast Asia and the Western Pacific [226]. Therefore, there is a significant need for effective and well-tolerated antiretroviral agents for the treatment of MDR HIV. Limited antiretrovirals are currently available for the treatment of MDR HIV-1 patients. In 2017, the PRESTIGIO Registry was established in order to collect clinical, virological and immunological monitoring data from people living with HIV with documented four-class drug resistance, specifically NRTIs, NNNRTIs, PIs and INSTIs. The residual susceptibility to specific antiretrovirals was evaluated, along with the validation of treatment and monitoring strategies in the studied population. Annual updates are scheduled [227].

### 4.1. HIV-1 Capsid Inhibitors

HIV is characterized by a conical capsid that encloses the viral RNA genome. The capsid is crucial for HIV-1 replication and plays an essential role in early and late stages of the viral lifecycle. In the first stages, after the fusion of viral and cellular membranes, the viral capsid is released into the host cell cytoplasm and dissociates in a process known as uncoating, which is tightly associated with the reverse transcription of the viral genome. The gag polyprotein, which is the precursor of the capsid protein, assembles at the plasma membrane to form immature non-infectious viral particles during the late stages of replication of the virus. After a maturation step by the viral protease, the capsid accumulates to form a fullerene-like conical shape characteristic of the mature infectious particle. Mutations affecting the uncoating process, or capsid assembly and maturation, have been demonstrated to prevent viral infectivity. Recent studies have focussed on the development of drugs acting as inhibitors of capsids (such as lenacapavir) [228]. These drugs are used in patients living with multidrug-resistant (MDR) HIV, who have limited antiretroviral regimen options that provide durable viral suppression.

The antiviral activity of lenacapavir against HIV was demonstrated in vitro against primary MDM and CD4+ T-lymphocytes, with picomolar mean half-maximum EC_50_ of 56 pM and 32 pM, respectively [25].

Lenacapavir, the first approved capsid inhibitor, has been shown to have a significantly higher potency than many existing antiretroviral drugs, with mean half-maximum EC_50_ of 105 pmol/L in the MT-4 human T cell line. The potency of lenacapavir was also confirmed in both primary human CD4+ T cells (EC_50_: 32 pmol/L) and MDM (EC_50_: 56 pmol/L). Moreover, lenacapavir has been demonstrated to be active against several HIV-1 subtypes and, although at a lower extent (15- to 25-fold less), against two HIV-2 isolates [229].

#### Lenacapavir

Lenacapavir (GS-6207, Sunlenca^®^) [230] is a small molecule that selectively targets the HIV-1 capsid protein (CA, p24), which is a structural component of the virus. It selectively binds to the interface of the two hexameric subunits of the HIV-1 capsid, resulting in the development or inhibition of the immature HIV-1 capsid; thus, it interferes with capsid-mediated HIV-1 nuclear uptake, viral assembly and release and capsid core formation during the HIV-1 lifecycle [231]. This is the first HIV-1 capsid protein inhibitor approved by the FDA for clinical use in heavily treatment-experienced patients with multidrug-resistant HIV-1 infection. On 22 December 2022, it received marketing authorisation by the FDA to treat adults with HIV whose HIV infections could not be successfully treated with other available treatments due to resistance, intolerance or safety considerations [232]. It is an HIV-1 capsid inhibitor with a multistage mechanism of action, indicated for multidrug-resistant HIV, and is being studied for PrEP. It may be taken orally daily or weekly or by subcutaneous injection twice a year. It demonstrated strong antiviral effectiveness in phase 1b clinical studies. Individuals with HIV-1 infection and MDR who received lenacapavir experienced a significant reduction in viral load compared to those who received a placebo at baseline [233]; cross-resistance to existing antiretroviral classes was not evidenced [204]. Injection site reactions are the most commonly reported reactions to this drug [226,234].

### 4.2. Monoclonal Antibodies

#### Ibalizumab

Ibalizumab (Trogarzo^®^; ibalizumab-uiyk) was the first monoclonal antibody (MAb) approved for the treatment of HIV-1 infection. As a CD4-directed, post-attachment inhibitor, ibalizumab is able to block HIV-1 entry into CD4 cells while preserving normal immune function. In particular, ibalizumab is a recombinant humanized immunoglobulin with a novel antiviral mechanism. Indeed, ibalizumab binds to the CD4 extracellular domain 1 at specific amino acid sites, blocking exposure of the V3 loop, consequently inhibiting the interaction of gp120 with the CXCR4 and CCR5 co-receptors [235,236,237,238,239,240]. This subsequently prevents viral fusion and entry into the CD4+ T cells [64]. 

In the US, it was approved for intravenous use in association with other antiretrovirals for the treatment of heavily treatment-experienced adults with multidrug-resistant HIV-1 infection failing their current antiretroviral regimen [241], and in the EU for the treatment of adults infected with multidrug-resistant HIV-1 infection for whom it was otherwise not possible to construct a suppressive antiviral regimen [242].

No data on the antiviral efficacy of ibalizumab in MDM are yet available. Nevertheless, this issue deserves careful investigations, considering, on one hand, the higher affinity of the viral envelopes of M-tropic HIV-1 isolates for the CD4 receptor expressed by macrophages (in comparison to those expressed by CD4+ T cells) [243,244,245,246] and, on the other hand, the enhanced interactions of M-tropic virus envelopes with the CCR5 or CXCR4 co-receptors in macrophages [243]. Taken together, these considerations raise the relevance to assess the activity of post-attachment inhibitors in MDM.

## 5. Long-Acting Drugs

To overcome the limitations of life-long daily regimen adherence, long-acting (LA) injectable antiretroviral drugs, nanoformulations, implants, vaginal rings, microarray patches and ultra-long-acting (ULA) prodrugs are now available or are in development [247]. First-generation LA cabotegravir, rilpivirine and lenacapavir injectables and a dapivirine vaginal ring are now in use [248]. In addition, the FDA has approved cabotegravir as oral tablets (VOCABRIA™) for use in combination with oral rilpivirine (Edurant^®^) [249] prior to initiating the intramuscular treatment regimen or as an oral bridging therapy for missed CABENUVA injections. Vocabria™ is given as one 30 mg tablet with one 25 mg tablet of Edurant^®^ once a day. Cabotegravir has a low potential to cause drug–drug interactions [250]. LA cabotegravir and rilpivirine have also demonstrated few and non-severe side effects in adolescents [251]. Albuvirtide is an LA HIV fusion inhibitor with limited drug–drug interactions and a fast onset time [252]. Lipid-based depots have also been reported for HIV infection. They are biocompatible, provide extended drug release and improve drug stability, making them suitable for systemic and localized treatment [253]. Surve et al. (2020) [254] developed an efavirenz and enfuvirtide co-loaded lipid-polymer hybrid nanoparticle system for sustained drug release and enhanced delivery to immune cells, such as T cells and macrophages. Recently, in vitro and in vivo studies on long-acting subcutaneous raltegravir injections and prodrugs have been described for the treatment of HIV [255]. Moreover, LA drug delivery systems (DDSs) are used for extended therapeutic release in order to improve regimen adherence and reduce toxicity. Cabenuva, Apretude and Sunlenca LA DDSs are safe and effective [256].

There is accumulating evidence on the therapeutic advantages of long-acting drugs, based on their better cell-mediated drug delivery even to areas of poor drug penetrance and to viral reservoirs [257]. It should be remarked that a relevant role in these improved properties of long-term formulations is played by MDM. Indeed, several studies have demonstrated that MDM can uptake and sequestrate large amounts of the antiviral drug, thus acting as Trojan Horses for drug delivery to circulating and tissue CD4+ T cells and viral reservoirs [258,259]. This mechanism is particularly active at the level of lymphoid organs, where macrophages and T cells are in close contact, allowing the drug passage to such major reservoirs of HIV infection.

In this regard, a recent study has highlighted the role of macrophages as a major depot for cabotegravir prodrug nanoformulations, showing that the nanoformuled pro-drug was efficiently taken up and retained for at least 30 days by MDM. In particular, cabotegravir as nanoformulation demonstrated up to 100-fold greater uptake and retention by MDM compared to unformulated cabotegravir. This enhanced and sustained PK profile was mainly imputable to an enhanced cellular uptake of the nanocrystals from the injection site, intracellular retention and nanocrystal stability in macrophages [260]. These data demonstrate the relevance of an enhanced and more efficient macrophage delivery and accumulation of intracellular tissue drug depots in these cells for optimizing long-term antiretroviral strategies. 

## 6. Chemoprophylaxis/Pre-Exposure Prophylaxis (PrEP)

Chemoprophylaxis is an important option for the prevention of HIV infection. Currently, the WHO recommends three options for PrEP: oral tenofovir (TDF)/emtricitabine PrEP available in fixed dose combination (FDC); the dapivirine vaginal ring; intramuscular long-acting cabotegravir (CAB-LA) [211,212,213]. The two drug combinations Truvada^®^ (TDF and emtricitabine) and Descovy^®^ (TAF and emtricitabine) are currently available for this purpose. They can be administered orally once daily to prevent HIV infection from occurring PrEP. The US FDA approved the prophylactic use of Truvada^®^ on 16 July 2012, and for Descovy^®^ in 2019 [261]. Descovy is approved for daily use for everyone, except those vulnerable to HIV through vaginal sex [262]. The safety profiles of currently available PrEP candidates for women of child-bearing potential, those in pregnancy or those who are breastfeeding have been recently reviewed [263].

Also regarding PrEP, it is relevant to take carefully into account the efficacy of the antiretroviral drugs used in this context in myeloid compartments, and particularly in MDM it is critical to ensure a successful PrEP. Indeed, it is currently well recognized that, during the early phases of HIV infection following sexual transmission, macrophages, together with dendritic cells, at the level of genital and rectal submucosal tissues, constitute the first cells targeted by HIV-1 infection, and thus also play a pivotal role in viral transmission [264,265,266].

## 7. Combined Therapies

Several combinations of anti-HIV therapies are used in therapy. These are summarized in Table 2. Through 5 years of follow-up, based on NCT02607930, NCT02607956, EudraCT 2015-004024-54 and EudraCT 2015-003988-10 clinical studies, bictegravir/emtricitabine/TAF maintained high rates of virologic suppression with no treatment-emergent resistance and rare drug discontinuations due to adverse events, confirming the durability and safety of this combination in people with HIV [267,268]. CABENUVA™ is an extended-release injectable formulation of cabotegravir and rilpivirine for concurrent administration and was developed by ViiV Healthcare and Janssen Pharmaceutica (Janssen) as a complete regimen for HIV infection. Based on the results of the ATLAS and FLAIR trials, the regimen was approved in Canada on 20 March, 2020, for the treatment of HIV-1 infection in adults to replace current antiretroviral therapy in patients who are virologically stable and suppressed [269]. Triumeq^®^ is a combination product containing abacavir, dolutegravir and lamivudine tablets, first approved on 1 September 2014, to treat HIV infection. In 2022, with the approval of Triumeq dispersible tablets for oral suspension, the indicated population was expanded to include paediatric patients weighing at least 10 kg [270]. Evotaz^®^ is a combination of the PI atazanavir and CYP inhibitor cobicistat; thus, it inhibits both CYP enzymes and proteases at the same time [271]. It was approved by the US FDA in January 2015. However, it is not effective in children under 18 [272,273].

## 8. Novel Therapeutic Strategies for the Treatment of HIV

One of the most explored cure strategies to overcome drug toxicity, drug–drug interactions or drug resistance related to cART is the ‘Shock-and-Kill’ or ‘Kick and Kill’ therapy, which aims to reactivate the latent HIV-1 proviruses and afterwards kill the virus-producing cells [274]. It consists of treatment with latency-reversing agents (LRAs) to induce HIV provirus expression, thus exposing these cells to killing by cellular immunity or apoptosis. LRAs are used to induce the transcription of the provirus in latently infected cells, thus enabling the reactivation of HIV. The activated cells are then killed by immune-mediated mechanisms or viral cytolytic effects, because they can express viruses or antigens. Although the viruses produced by the reactivated latently infected cells might infect new cells, current treatments such as ART can prevent most uninfected cells from being infected. Current efforts to allow the ‘Shock-and-Kill’ strategy are aimed at the development of better combinations of LRAs to produce the wide and robust induction of the HIV provirus and enhancing the elimination of cells where replication has been reactivated by targeted immune modulation. However, the clinical trials regarding this strategy refer only to short periods because of toxicity concerns of LRAs. The short-term effects on the reservoir’s size may have been too small to detect in clinical trials. The ‘shock and kill’ strategy with vectored immunoprophylaxis (VIP) has been suggested to control HIV infection and to indicate the most appropriate time for a short-term LRAs treatment [275]. Roda et al. (2021) [276] developed a model to evaluate the effects of LRAs on HIV infection in the brain and concluded that the “shock and kill” strategy can eradicate the latent reservoir of brain macrophages. An alternative strategy for HIV treatment is ‘lock and block’ intervention, which involves preventing re-emergence of the virus from latently infected cells, thus inhibiting transcription of the provirus and preventing virus spread or disruption of the HIV provirus genome by genome editing [277]. HIV-1-specific broadly neutralizing monoclonal antibodies (bNAbs) have also been widely investigated in recent years. bNAbs provide a novel intervention for the control of HIV through targeting the HIV Env, facilitating the clearance of infected cells and boosting immune responses [278]. HIV-1 therapy with single or dual bNAbs has shown viral escape, indicating that at least a triple bNAb therapy may be needed for robust suppression of viremia [279,280]. The role of bNAbs in the “shock and kill” approach has been reviewed by Gunst et al. (2020) [281]. LRAs are used to reverse the provirus silenced in latently infected cells, and then reactivated cells are cleared by host immunity. However, such activation strategies have shown only limited success. A combination of LRAs and bNAbs is considered a promising approach for HIV-1 eradication [282]. 

In light of the central role of monocytes and macrophages as HIV reservoirs, persisting even under cART, the effect of LRA should be carefully verified in these cellular compartments. The observation that LRAs can reactivate latently infected macrophages in the CNS, leading to increased immune activation and inflammatory responses in SIV models, [283] underlines the potential risks that LRAs could worsen macrophage-mediated pathologies. Moreover, CTL-mediated killing activity against HIV-infected macrophages can increase inflammation [284]. In light of this, strategies to target the macrophage reservoir and minimize the exacerbated activation need to be further investigated, together with T cell-centric cure strategies.

Recently, the combination of a CRISPR activation (CRISPRa) system with gRNA-V, the truncated Bid (tBid)-based suicide gene strategy and CD3-retargeted adenovirus (Ad) delivery vectors, in an all-in-one targeted shock-and-kill gene therapy approach has been reported with the aim of the specific elimination of latently HIV-1-infected cells [285].

Chimeric antigen receptor (CAR) technology has been suggested as a potential therapy to achieve a sterilizing cure for human immunodeficiency virus (HIV) infection, hoping to “kill” the virus completely after “shock” treatment [286]. However, translation of this technology from the haematological malignancy field to the HIV scenario is not easy, as many challenges have appeared along the way that hinder the consolidation of CAR-T cells as a speculative therapy [287,288]. Furthermore, it should be carefully considered that CAR therapies are currently, predominantly targeted at T cells [289], with active, replicating viruses, and they most likely will not aid in the eradication of viruses from macrophages, particularly those in a latent state.

## 9. Other Activities of Antiretroviral Drugs

Several other activities have been reported for antiretroviral drugs, including antitumor activity. Reverse transcriptase inhibitors were shown to modulate cell growth and differentiation across various cancer types [290]. Moreover, ritonavir, atazanavir and lopinavir have been demonstrated to reduce the proliferation of schwannoma and grade I meningioma cells [291], whereas nevirapine has been demonstrated to be capable of lowering cell growth and promoting differentiation in acute myeloid leukemia cells and primary blasts [292]. Moreover, treatment with nevirapine and efavirenz determined the reversible inhibition of cell proliferation in an undifferentiated thyroid carcinoma cell line with high endogenous RT activity [293]. Recent studies report that antiretroviral drugs are able to modulate the expression of human endogenous retroviruses (HERVs), which exhibit upregulation across various tumour types, with a particularly pronounced presence in melanoma. Specifically, lamivudine, doravirine and cabotegravir are able to downregulate the expression of human endogenous retroviruses (HERVs), inhibit cell growth and reduce invasive capabilities in melanoma cell lines [294]. Our investigation demonstrates that antiretrovirals belonging to the NRTI, NNRTI and INSTI classes can downregulate the expression of HERV-K genes in four distinct melanoma cell lines, thereby limiting the aggressive potential of these cells. Specifically, cabotegravir, a novel antiretroviral drug targeting integrase, proves to be a potent tool for inhibiting cell growth and invasion potential through various mechanisms in different cell lines. Beyond its antiretroviral effects, cabotegravir holds promise for potential use in conjunction with other chemotherapeutic agents, as it exhibits the ability to induce cell growth inhibition, apoptosis and ferroptosis and hinders the migration and adhesion of melanoma cells. Lastly, given the significance of the PD-1/PD-L1 pathway as a crucial target in cancer immunotherapy, chemotherapeutic molecules with the ability to downregulate PD-L1 expression in tumour cells hold promise for enhancing the immune response. Studies on CD133-positive melanoma stem cells demonstrated that nevirapine and efavirenz downregulate HERV-K activity concurrent with a decrease in the proliferative rate and CD133 expression [295]. Recently, the role of abnormal HERVs activity has been described in relation to neurodevelopmental disorders [296]. Giovinazzo et al. suggested the use of antiretroviral drugs as an innovative approach to treat aggressive tumours in combination with chemotherapeutic/radiotherapy regimens [297]. Nelfinavir and lopinavir/ritonavir are FDA-approved HIV-protease inhibitors that inhibit SARS-CoV-2 replication in vitro [149]. Moreover, nelfinavir is under study for its anticancer properties, either as a single drug or in combination with chemoradiotherapy, thus being suggested as a suitable candidate for drug repurposing for cancer. 

## 10. Vaccines

While various treatment and prevention methods exist, including antiretroviral therapy and non-vaccine approaches, developing an effective vaccine remains the most crucial and cost-effective solution to combat the HIV epidemic. Despite significant advancements in HIV research, the HIV vaccine field has faced numerous challenges, and only one clinical trial has demonstrated a modest level of efficacy. Pre-clinical vaccine development is carried out using the non-human primate model (NHP) of HIV infection [298,299]. NHP models offer valuable insights into potential preventive strategies for combating HIV, and they play a vital role in informing and guiding the development of novel vaccine candidates before they can proceed to human clinical trials. Recent clinical trials have demonstrated that anti-HIV-1 bNAbs are able to control viremia in people living with HIV and also to improve the host’s humoral and cellular immune response. Such vaccinal effects, particularly the induction of HIV-1-specific CD8+ T cell responses, were observed after treatment with two potent bNAbs (3BNC117 and 10-1074), alone or in combination with latency-reversing agents (LRA) [300,301].

## 11. Clinical Studies in HIV

Several clinical studies regarding HIV treatment are underway or have been recently completed. They are summarized in Table 3. However, Zhou et al. (2023) [302] have recently suggested that US females are underrepresented in phase 3 HIV-1 clinical trials. Some drugs are not yet approved, such as MK-8527 [116,303], a new NRTTI belonging to the same class as islatravir (NCT0604550).

## 12. Conclusions

AIDS is caused by infection with the HIV virus. Although treatments against HIV infection are available, AIDS remains a serious disease that causes many deaths annually. cART, also known as HAART, is a treatment with a combination of several antiretroviral drugs that block multiple stages in the virus replication cycle. However, as more HIV patients start cART, the emergence of HIV drug resistance is inevitable. cART may also cause viral rebound after treatment discontinuation. Hence, people with HIV have to take cART life-long, and drug toxicity, drug–drug interactions or drug resistance are just some of the challenges of managing the HIV pandemic, and they stress the need for an HIV-1 cure. HIV preferentially infects activated CD4+ T cells. Current antiretroviral therapy cannot eradicate the virus, and the viral infection of other cells, such as macrophages, may contribute to viral persistence during antiretroviral therapy. Indeed, in addition to cell-free virus infection, macrophages can also become infected when engulfing infected CD4+ T cells as innate immune sentinels. The persistence of monocyte/macrophage reservoirs in HIV+ individuals on cART should be taken carefully into account since, besides contributing to pathologies, it can represent a relevant barrier against the achievement of a HIV cure. In light of this, improving drug efficacies in heterogeneous target cells, including macrophages, is crucial to finally achieve HIV eradication in infected individuals. 

## Figures and Tables

**Table 1:** Drugs currently used for HIV therapy.

Structure	Name	Class
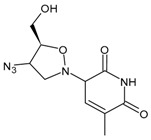	Zidovudine (AZT, ZVD)	NRTIs (used in combination)
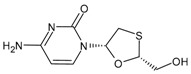	Lamivudine (3TC or LAM)	NRTIs (used in combination)
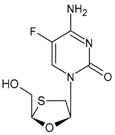	Emtricitabine (FTC)	NRTIs (used in combination)
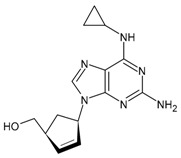	Abacavir (ABC)	NRTIs (used alone or in combination)
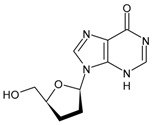	Didanosine (ddI)	NRTIs (used in combination)
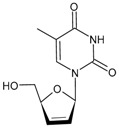	Stavudine (d4T)	NRTIs (used in combination)
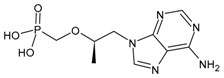	Tenofovir (TFV)	NRTIs (used alone or in combination)
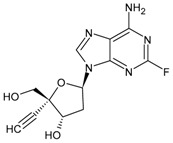	Islatravir (ISL)	NRTIs (used in combination)
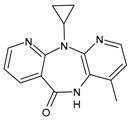	Nevirapine (NVP)	NNRTIs (used in combination)
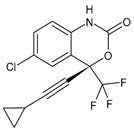	Efavirenz (EFV)	NNRTIs (used in combination)
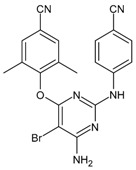	Etravirine (ETR)	NNRTIs (used in combination)
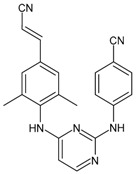	Rilpivirine (RPV)	NNRTIs (used in combination)
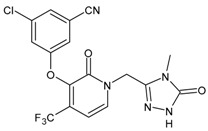	Doravirine (DOR)	NNRTIs (used in combination)
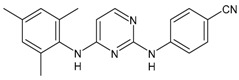	Dapivirine (DPV)	NNRTIs (used alone)
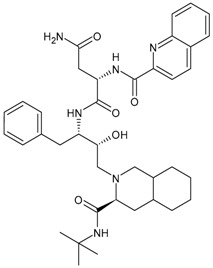	Saquinavir (SQV)	PIs (used in combination)
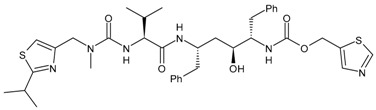	Ritonavir (RTV or RIT)	PIs and CYP inhibitor (used in combination)
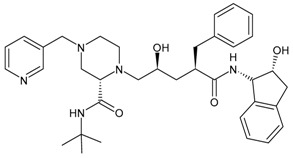	Indinavir (IDV)	PIs (used alone or in combination)
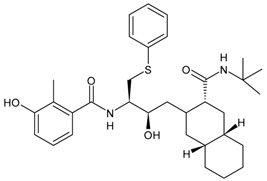	Nelfinavir (NFV)	PIs (used in combination)
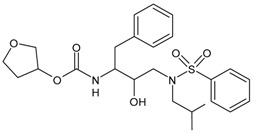	Amprenavir (APV)	PIs (used in combination)
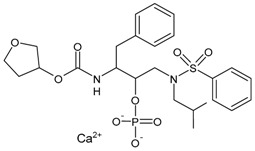	Fosamprenavir (prodrug of amprenavir) (FPV)	PIs (used in combination)
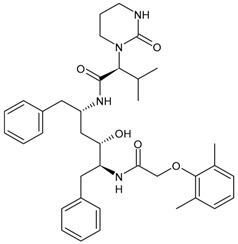	Lopinavir (LPV)	PIs (used in combination)
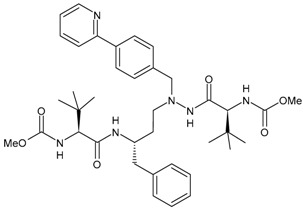	Atazanavir (ATZ or ATV)	PIs (used in combination)
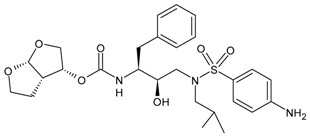	Darunavir (DRV)	PIs (used alone or in combination)
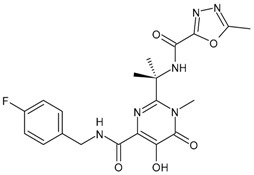	Raltegravir (RAL)	INSTIs (used in combination)
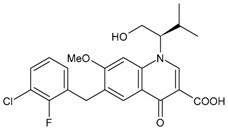	Elvitegravir (EVG)	INSTIs (used in combination)
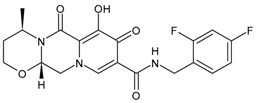	Dolutegravir (DTG)	INSTIs (used alone or in combination)
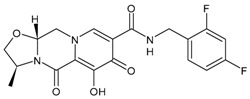	Cabotegravir (CAB)	INSTIs (used alone or in combination)
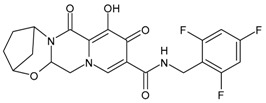	Bictegravir (BIC)	INSTIs (used in combination)
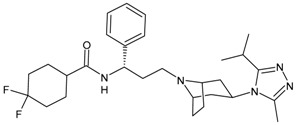	Maraviroc (MVC)	CCR5 antagonist (used in combination)

**Table 2:** Combination therapies for the treatment of HIV.

Combinations (Approval Date)	TDF	TAF	Emtricitabine (FTC)	Efavirenz (EFV)	Rilpivirine (RPV)	Dolutegravir (DTG)	Elvitegravir (EVG)	Cobicistat (COBI)	Bictegravir (BIC)	Darunavir (DRV)	Doravirine (DOR)	Cabotegravir (CAB)	Zidovudine (AZT, ZVD)	Lamivudine (LAM, 3TC)	Abacavir (ABC)
Truvada^®^ 2004	+		+												
Atripla^®^ 2006	+		+	+											
Complera^®^ (US) Eviplera^®^ (EU) 2011	+		+		+										
Stribild^®^ or QUAD pill 2012	+		+				+	+							
Descovy^®^ 2019		+	+												
Genvoya^®^ 2015		+	+				+	+							
Odefsey^®^ 2016		+	+		+										
Biktarvy^®^ 2018		+	+						+						
Symtuza^®^ 2018		+	+					+		+					
Rezolsta^®^ 2014								+		+					
Dovato^®^ 2019						+								+	
CABENUVA™ 2020					+							+			
Epzicom^®^ (US), *Kivexa* (Korea) 2011														+	+
Combivir^®^ 1997													+	+	
Trizivir^®^ 2000													+	+	+
Triumeq^®^ 2014						+								+	+
Delstrigo^®^ 2018	+										+			+	

The sign “+” indicates that the drug is present in the corresponding combination therapy reported.

**Table 3:** Clinical studies related to HIV infection.

Name of the Clinical Trial	ClinicalTrials.gov Identifier	Phase	Actual Study Start Date	Estimated Study Completion Date or Completion Date
A Phase 2 Randomized, Open-Label, Active-Controlled Study Evaluating the Safety and Efficacy of an Oral Weekly Regimen of Islatravir in Combination with Lenacapavir in Virologically Suppressed People with HIV	NCT05052996	Phase 2	5 October 2021	November 2027
A Study of Doravirine/Islatravir (DOR/ISL, MK-8591A) for the Treatment of Human Immunodeficiency Virus 1 (HIV-1) Infection in Participants Who Previously Received DOR/ISL (MK-8591A-054)	NCT05766501	Phase 2	17 March 2023	14 January 2026
A Phase 3 Randomized, Active-Controlled, Double-Blind Clinical Study to Evaluate the Antiretroviral Activity, Safety, and Tolerability of Doravirine/Islatravir Once-Daily in HIV-1-Infected Treatment-Naïve Participants	NCT04233879	Phase 3	28 February 2020	3 March 2025
Safety of and Immune Response to Dolutegravir in HIV-1-Infected Infants, Children, and Adolescents	NCT01302847	Phase 1/2	20 April 2011	20 January 2024
A Phase 2 Randomized, Open-Label, Active-Controlled Study Evaluating the Safety and Efficacy of an Oral Weekly Regimen of Islatravir in Combination with Lenacapavir in Virologically Suppressed People with HIV	NCT05052996	Phase 2	5 October 2021	November 2027
A Study of Doravirine/Islatravir (DOR/ISL, MK-8591A) for the Treatment of Human Immunodeficiency Virus 1 (HIV-1) Infection in Participants Who Previously Received DOR/ISL (MK-8591A-054)	NCT05766501	Phase 2	17 March 2023	14 January 2026
Effect of PCSK9 Inhibition on Cardiovascular Risk in Treated HIV Infection (EPIC-HIV Study) (EPIC-HIV)	NCT03207945	Phase 3	30 April 2018	July 2025
Pre-Exposure Prophylaxis Study of Lenacapavir and Emtricitabine/Tenofovir Alafenamide in Adolescent Girls and Young Women at Risk of HIV Infection (PURPOSE 1)	NCT04994509	Phase 3	30 August 2021	July 2027
Study of Lenacapavir for HIV Pre-Exposure Prophylaxis in People Who Are at Risk for HIV Infection (PURPOSE 2)	NCT04925752	Phase 3	28 June 2021	April 2027
Italian Registry of HIV-1-Infected Patients with Drug-RESistant Virus to Reverse Transcriptase Inhibitors, InteGrasE and Viral Protease. (PRESTIGIO)	NCT04098315	Observational	14 December 2017	31 December 2028
A Randomised Placebo Controlled Trial of ART Plus Dual Long-acting HIV-specific Broadly Neutralising Antibodies (bNAbs). (RIO)	NCT04319367	Phase 2	17 May 2021	31 March 2025
Effects of Switching From ATRIPLA™ (Efavirenz, Tenofovir, Emtricitabine) to MK-1439A (Doravirine, Tenofovir, Lamivudine) in Virologically-Suppressed Participants (MK-1439A-028)	NCT02652260	Phase 2	4 March 2016	29 February 2024
Safety and Pharmacokinetic Study of Oral MK-8527 QM in Participants at Low-Risk for HIV-1 Infection (MK-8527-007)	NCT06045507	Phase 2	8 November 2023	18 February 2025
A Clinical Trial of STP0404 in Treatment-Naïve Adults with HIV-1 Infection	NCT05869643	Phase 2	23 May 2023	13 May 2024
HIV-1-Infected Patients, Phase II Trial, Dual Combination Doravirine/Raltegravir Open Label (DORAL)	NCT04513626	Phase 2	15 September 2020	30 October 2024
Doravirine for Persons with Excessive Weight Gain on Integrase Inhibitors and Tenofovir Alafenamide	NCT04636437	Phase 4	20 May 2021	31 October 2024
CAR-T Cells for HIV Infection	NCT04648046	Phase 1/2	1 March 2021	31 December 2027
Study to Evaluate Pharmacokinetic and Safety of Albuvirtide between Intravenous Drip and Intravenous Injection	NCT05206019	Phase 1	16 February 2022	Completed 2 May 2022
An Efficacy, Safety, and Tolerability Study Comparing Dolutegravir (DTG) Plus Lamivudine (3TC) with Dolutegravir Plus Tenofovir/Emtricitabine in the Treatment of Naïve HIV Infected Participants (Gemini 2)	NCT02831764	Phase 3	18 July 2016	Completed 26 June 2022
An Efficacy, Safety, and Tolerability Study Comparing Dolutegravir Plus Lamivudine with Dolutegravir Plus Tenofovir/Emtricitabine in Treatment naïve HIV Infected Subjects (Gemini 1)	NCT02831673	Phase 3	21 July 2016	Completed 15 August 2022
Switch Study to Evaluate Dolutegravir Plus Lamivudine in Virologically Suppressed Human Immunodeficiency Virus Type 1 Positive Adults (TANGO)	NCT03446573	Phase 3	18 January 2018	Completed 3 May 2022
Islatravir (MK-8591) with Doravirine and Lamivudine in Participants Infected with Human Immunodeficiency Virus Type 1 (MK-8591-011)	NCT03272347	Phase 2	27 November 2017	Completed 9 March 2022
Study to Evaluate the Safety and Efficacy of Bictegravir/Emtricitabine/Tenofovir Alafenamide versus Dolutegravir + Emtricitabine/Tenofovir Alafenamide in Human Immunodeficiency Virus (HIV-1)-Infected, Antiretroviral Treatment-Naive Adults	NCT02607956	Phase 3	11 November 2015	Completed 5 July 2021
Study to Evaluate the Safety and Efficacy of Bictegravir/Emtricitabine/Tenofovir Alafenamide versus Abacavir/Dolutegravir/Lamivudine in Human Immunodeficiency Virus-1 (HIV-1)-Infected, Antiretroviral Treatment-Naive Adults	NCT02607930	Phase 3	13 November 2015	Completed 2 July 2021
Safety and Efficacy of Switching from Dolutegravir and ABC/3TC or ABC/DTG/3TC to B/F/TAF in HIV-1-Infected Adults Who Are Virologically Suppressed	NCT02603120	Phase 3	11 November 2015	Completed 23 October 2019
Study to Evaluate the Safety and Efficacy of Switching from Regimens Consisting of Boosted Atazanavir or Darunavir Plus Either Emtricitabine/Tenofovir or Abacavir/Lamivudine to Bictegravir/Emtricitabine/Tenofovir Alafenamide in Virologically Suppressed HIV-1-Infected Adults	NCT02603107	Phase 3	20 November 2015	Completed 23 December 2019
Safety and Efficacy of Switching to an FDC of B/F/TAF From E/C/F/TAF, E/C/F/TDF, or ATV+RTV+FTC/TDF in Virologically Suppressed HIV-1-Infected Women	NCT02652624	Phase 3	19 February 2016	Completed 26 November 2018
Trial to Assess the Continued Safety of and Adherence to a Vaginal Ring Containing Dapivirine in Women	NCT02858037	Phase 3	18 July 2016	Completed 10 October 2018

## Data Availability

Not applicable.
